# Transmission probability of gas molecules through porous layers at Knudsen diffusion

**DOI:** 10.1007/s10665-023-10308-0

**Published:** 2023-12-07

**Authors:** Wolfgang Macher, Yuri Skorov, Günter Kargl, Sunny Laddha, Stephan Zivithal

**Affiliations:** 1 Space Research Institute, Austrian Academy of Sciences, Schmiedlstraße 6, 8042 Graz, Austria; 2 Max Planck Institute for Solar System Research, Justus-von-Liebig-Weg 3, 37077 Göttingen, Germany; 3 Institute of Geophysics and Extraterrestrial Physics, Techn. Univ. Braunschweig, Mendelssohnstraße 3, 38106 Brunswick, Germany

**Keywords:** Knudsen diffusion, Monte Carlo simulation, Porous media, Rarified gas flow, 60J60, 76R50, 76P99, 76S99

## Abstract

Gas flow through layers of porous materials plays a crucial role in technical applications, geology, petrochemistry, and space sciences (e.g., fuel cells, catalysis, shale gas production, and outgassing of volatiles from comets). In many applications the Knudsen regime is predominant, where the pore size is small compared to the mean free path between intermolecular collisions. In this context common parameters to describe the gas percolation through layers of porous media are the probability of gas molecule transmission and the Knudsen diffusion coefficient of the medium. We show how probabilistic considerations on layer partitions lead to the analytical description of the permeability of a porous medium to gas flow as a function of layer thickness. The derivations are made on the preconditions that the molecule reflection at pore surfaces is diffuse and that the pore structure is homogenous on a scale much larger than the pore size. By applying a bi-hemispherical Maxwell distribution, relations between the layer transmission probability, the half-transmission thickness, and the Knudsen diffusion coefficient are obtained. For packings of spheres, expressions of these parameters in terms of porosity and grain size are derived and compared with former standard models. A verification of the derived equations is given by means of numerical simulations, also providing evidence that our analytical model for sphere packing is more accurate than the former classical models.

## Introduction

Models of gas flow through porous media can be categorized according to the assumptions made on the properties of the solid material and the gas species flowing through the pores. For inert gases the models essentially differ in the way the pore structure and the gas collisions are represented. A systematic approach to the description of rarefied gas flow through porous media dates back to Knudsen’s work on gas flow through cylinders [1]. The often used application to porous media regard the pores in the medium as a collection of straight cylindrical filaments which are distributed arbitrarily (with varying orientations) throughout the medium. More sophisticated models were considered later by Derjaguin [2, 3] and Asaeda et al. [4], which are based on packing of spheres. These approaches are also used in the present article to link the transmission probability with other parameters used to describe the gas diffusion through the porous medium. Recent works apply numerical methods, in particular Direct Simulation Monte Carlo (DSMC) [5, 6], to take complicated pore shapes into account [7, 8].

Here we focus on the gas density range where the pore size is much smaller than the mean free path between intermolecular collisions.

This Knudsen regime plays an important role in material sciences, especially in relation with adsorption [9, 10] and catalytic processes [11], which are the basis of many technical applications (e.g., fuel cells and catalysts for a great variety of chemical products). It was studied also in the context of shale gas extraction [12], which became more important for the worldwide energy supply in the last decades. Furthermore, the Knudsen pressure regime is of great relevance in space sciences. In particular, it prevails at processes of outgassing from comet surfaces [13–15]. At sufficiently low pressures the so-called Knudsen diffusion determines the gas flow. In contrast to viscous flow, where the pressure gradient causes the upstream molecules to impart, on the average, a momentum to the downstream molecules, the situation is different for Knudsen diffusion: Because the molecule motion in the pore volume occurs without intermolecular collisions, a net flow can only be the result of spatial variations of gas density or temperature (mean molecule speed). Assuming diffuse or specular reflection of gas molecules at the pore walls, the Knudsen diffusion through a layer of porous material depends only on the pore structure and the density/velocity distribution of the gas molecules. Since the motion of gas molecules through the medium is a stochastic process, the steady-state flow through the layer can be analyzed on the basis of probabilistic considerations, as elaborated in this article for the special case of planar layers. Among other relations it yields the fraction ${{\mathcal {T}}}(z)$ of molecules reaching the distance *z* from the surface in the porous medium (the others return to the surface before having reached this depth). ${{\mathcal {T}}}(z)$ can also be regarded as the molecule transmission probability of a layer of thickness *z*, i.e., the probability that a molecule entering the pores at one side of the layer will exit at the other side. Former authors like Gundlach et al. [13], Skorov and Blum [16] used the heuristic formula

1 $$\begin{aligned} {{\mathcal {T}}} = \frac{a}{1+z/b} \end{aligned}$$

with constant medium parameters *a* and *b*. The probability approach described in the following enables us a relatively simple justification of this formula.

**Fig. 1:**
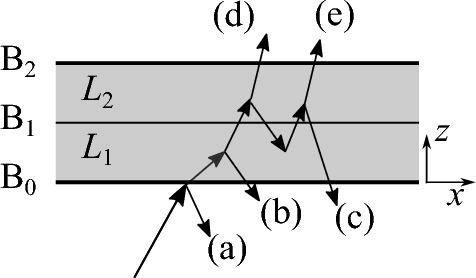
Schematic of possible reflection and transmission scenarios of a gas molecule on its path through a two-layer porous medium with layer boundaries $\textrm{B}_0$, $\textrm{B}_1$, and $\textrm{B}_2$. Different paths (**a**)–(**e**) are shown as examples

For this purpose the scenario illustrated in Fig. 1 is investigated, which consists of an infinite planar layer of porous medium divided into two sub-layers. Most of the molecules entering the layer at one side (boundary $\textrm{B}_0$) leave the medium only after long erratic paths, since they are reflected many times at the pore wall in the medium. The fraction of molecules penetrating to a certain distance *z* from the surface $\textrm{B}_0$ decreases with increasing *z*. Therefore, when the inflow at the opposite sides of the layer is different, a corresponding change of the gas density prevails across the layer under steady-state conditions. Neglecting intermolecular collisions and providing isothermal conditions (implying constant mean molecular speed across the sample), the net molecular flux divided by the magnitude of the gas density gradient is a constant $D^\textrm{K}$, the Knudsen diffusion coefficient.

In many applications it is advantageous to use ${{\mathcal {T}}}$ to describe the gas molecules throughput through a layer [13, 14]. In other circumstances, in particular when the gas flux or density (as it develops throughout the medium) is of interest, the Knudsen diffusion coefficient $D^\textrm{K}$ is sought [15, 17, 18]. One of the possible approaches to link these parameters is based on a bi-hemispheric Maxwell model of the velocity distribution, which was also applied by Asaeda et al. [4] for gas flow through packings of spherical grains. They expressed $D^\textrm{K}$ as a function of the grain diameter $d_\textrm{g}$ and the porosity $\epsilon$ of the packing. Further details are discussed in Sect. 4.

**Fig. 2:**
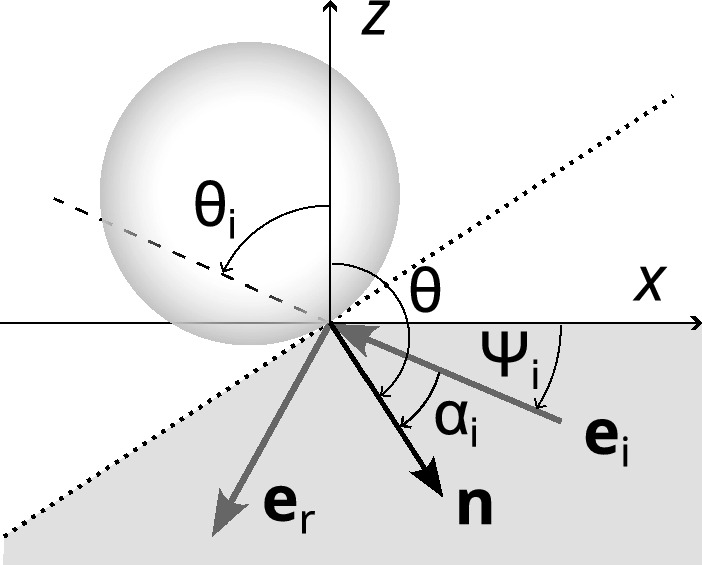
Molecule reflection at the surface of a sphere. The *x* axis is chosen in the plane spanned by the *z*-axis and the normal of the tangential plane (dotted) at the chosen collision point. The *y*-axis points away from the observer. The shaded area indicates the possible directions of incidence $-\textbf{e}_\textrm{i}$ of molecules with $v_z>0$ at the given point on the sphere. $\textbf{e}_\textrm{i}$ and the direction of reflection, $\textbf{e}_\textrm{r}$, need not be in the shown cross section (*xz*-plane)

Section 2 starts with an investigation of the transmission properties of two layers of constant porosity, which is related to the Knudsen diffusion constant in Sect. 3, and compared with models for $D^\textrm{K}$ in Sect. 4. Section 5 gives a validation of the derived analytical models by means of numerical simulations on the basis of two independent approaches. The most important symbols used in this article are illustrated in the Figs. 1 and 2 or listed in Table A1 for convenience.

## Transmission through two layers

The analysis of the interdependence of transmission probability and diffusion coefficient requires some basic relations on the transmission and reflection properties of the porous layer. For this purpose the medium is divided into two sub-layers of thickness $L_1$ and $L_2$ as shown in Fig. 1, with outer boundaries $\textrm{B}_0$ and $\textrm{B}_2$ and interfacial boundary $\textrm{B}_1$. We assume that the medium in the layers is homogeneous, with porosity $\epsilon$. Each boundary is considered as a planar medium cross section. For a packing of spherical grains of diameters $d_\textrm{g}$ it means that the spherical sections projecting beyond $\textrm{B}_0$ and $\textrm{B}_2$ are removed. This plane surface is different from surfaces of real-world packings where the surface consists of spherical surface sections. However, the plane-cut assumption facilitates a clear distinction between the immediate reflection of incident gas molecules at the surface boundary and the reflection by their coming back to the surface after having entered the layer(s) pores. We further assume that the cross-section area porosity (area of pore cross section divided by total cross-section area) equals the volume porosity of the whole layer. This precondition is guaranteed by the main assumption maintained throughout this article, which is the homogeneous distribution of grains in each sub-layer. More precisely, the distribution of the orientations of pore surface area elements does not depend on location (*x*, *y*, *z*) on a macroscopic scale much greater than the typical grain/pore size.

As a gas molecule traverses two adjacent layers or is reflected by them, it may cross the boundary between the layers several times before exiting the medium on either side. Possible path examples are indicated in Fig. 1: (a) Immediate reflection at the surface $\textrm{B}_0$, which occurs with probability $1-\epsilon$, provided the above-mentioned preconditions. (b) The gas molecule enters the layer 1, moves through its pores, and comes out after one or several reflections at the pore walls. (c) The molecule also enters layer 2 but escapes again through the surface $\textrm{B}_0$ after having moved forth and back between the layers, crossing the boundary $\textrm{B}_1$ an even number of times. (d) &(e) The molecule does finally leave through boundary $\textrm{B}_2$, which may happen after crossing $\textrm{B}_1$ one or several (an odd number of) times. The cases (a), (b), and (c) are reflections at the two-layer structure, whereas (d) and (e) are transmission scenarios which may be much more involved than shown in Fig. 1 (many crossings of $\textrm{B}_1$ are possible). Let ${{\mathcal {T}}}_i$ be the transmission probability of layer *i*, and ${{\mathcal {R}}}_i$ the corresponding layer reflection probability, under the assumption that the molecule has entered the layer pores (has not been reflected at the grain cut areas of the entrance surface $\textrm{B}_0$). We consider an inert solid matrix without adsorption processes at the pore surface. So ${\mathcal {T}}_i+{\mathcal {R}}_i=1$ since no sinks or sources are present. The probability of a molecule entering the pores through the boundary $\textrm{B}_0$ equals $\epsilon$. So the probabilities of the paths (a)–(e) are $1-\epsilon$, $\epsilon {{\mathcal {R}}}_1$, $\epsilon {{\mathcal {T}}}_1 {{\mathcal {R}}}_2 {{\mathcal {T}}}_1$, $\epsilon {{\mathcal {T}}}_1 {{\mathcal {T}}}_2$, and $\epsilon {{\mathcal {T}}}_1 {{\mathcal {R}}}_2 {{\mathcal {R}}}_1 {{\mathcal {T}}}_2$, respectively. The probability of a molecule incident onto $\textrm{B}_0$ to traverse both layers after moving forth and back between the layers, crossing $\textrm{B}_1$ $(2n+1)$ times, is $\epsilon {{\mathcal {T}}}_1({{\mathcal {R}}}_2 {{\mathcal {R}}}_1)^n{{\mathcal {T}}}_2$. The total transmission probability ${{\mathcal {T}}}_\textrm{t}$ is the sum over these possibilities

2 $$\begin{aligned} {{\mathcal {T}}}_\textrm{t}&= \epsilon \,{{\mathcal {T}}}, \end{aligned}$$

3 $$\begin{aligned} {{\mathcal {T}}}&= \sum _{n=0}^\infty {{\mathcal {T}}}_1({{\mathcal {R}}}_2 {{\mathcal {R}}}_1)^n{{\mathcal {T}}}_2 = \frac{{{\mathcal {T}}}_1{{\mathcal {T}}}_2}{1-{{\mathcal {R}}}_1 {{\mathcal {R}}}_2}. \end{aligned}$$

Here ${{\mathcal {T}}}$ denotes the transmission probability under the condition that the molecule crosses the boundary at a pore opening. The probability that the molecule is reflected by the two-layer structure can be calculated by a similar sum of path probabilities, or simply as

4 $$\begin{aligned} {{\mathcal {R}}}_\textrm{t}&= 1-{{\mathcal {T}}}_\textrm{t} = 1-\epsilon +\epsilon {{\mathcal {R}}}, \end{aligned}$$

5 $$\begin{aligned} {{\mathcal {R}}}&= 1-{{\mathcal {T}}} = \frac{{{\mathcal {R}}}_1+{{\mathcal {R}}}_2-2{{\mathcal {R}}}_1 {{\mathcal {R}}}_2}{1-{{\mathcal {R}}}_1 {{\mathcal {R}}}_2} = \frac{{{\mathcal {R}}}_1{{\mathcal {T}}}_2+{{\mathcal {R}}}_2{{\mathcal {T}}}_1}{1-{{\mathcal {R}}}_1 {{\mathcal {R}}}_2}, \end{aligned}$$

where ${{\mathcal {R}}}$ is the reflection probability associated with the two-sub-layer structure under the condition that the molecule falls onto an open pore. Dividing reflection by transmission probability proves that this fraction is an additive quantity

6 $$\begin{aligned} \frac{{{\mathcal {R}}}}{{{\mathcal {T}}}}= \frac{{{\mathcal {R}}}_1{{\mathcal {T}}}_2+{{\mathcal {R}}}_2{{\mathcal {T}}}_1}{{{\mathcal {T}}}_1{{\mathcal {T}}}_2} = \frac{{{\mathcal {R}}}_1}{{{\mathcal {T}}}_1} + \frac{{{\mathcal {R}}}_2}{{{\mathcal {T}}}_2}. \end{aligned}$$

Thus, by dividing the layer in more and more sub-layers (with equal properties), we recognize that ${{\mathcal {R}}}/{{\mathcal {T}}}$ is proportional to the layer thickness *L*. So it can be interpreted as a measure of resistance of the layer to gas flow, and ${{\mathcal {R}}}/({{\mathcal {T}}}\,L)$ must be constant (independent of *L*). In other words, the layer reflection and transmission probabilities, ${{\mathcal {R}}}(L)$ and ${{\mathcal {T}}}(L)$, which are functions of the layer thickness *L*, can be used to define the quantity

7 $$\begin{aligned} L_\textrm{h} = \frac{L\,{{\mathcal {T}}}(L)}{{{\mathcal {R}}}(L)}, \end{aligned}$$

which is independent of *L* and characterizes the medium itself. By substituting ${{\mathcal {T}}}=1-{{\mathcal {R}}}$ in (7) we obtain after rearrangement

8 $$\begin{aligned} {{\mathcal {R}}}&= \frac{L/L_\textrm{h}}{1+L/L_\textrm{h}}, \end{aligned}$$

9 $$\begin{aligned} {{\mathcal {T}}}&= \frac{1}{1+L/L_\textrm{h}}. \end{aligned}$$

These equations reveal that $L_\textrm{h}$ is the layer thickness for which half the gas molecules incident on open pores traverse the layer through the pores, the other half returning to the surface boundary where they entered. The last result can be rephrased: among the gas molecules entering the layer, the fraction $1/(1+z/L_\textrm{h})$ reaches the distance *z* from the surface. It does not decrease exponentially with *z*, which is a result of the fact that molecules reflected backwards are not removed from the flow process (as it occurs for molecule and light ray attenuation due to absorption), but may come again to the same position *z* after other reflections at the pore wall and then may advance further into the medium. The total transmission probability ${{\mathcal {T}}}_\textrm{t}$ is obtained from (9) by taking into account (2). It is, in fact, of the form (1) discussed above, showing the meaning of $a=\epsilon$ and $b=L_\textrm{h}$. Since in reality the boundary of a sample is not a plane cut, the parameters *a* and *b* may differ from these values, allowing for a modification of the reflection at the layer boundary in accordance with its uneven surface structure.

## Relation between transmission property and diffusion coefficient

A relation between $L_\textrm{h}$ and $D^\textrm{K}$ can be found by explicitly analyzing the flow contributions of the gas molecules moving forwards and backwards (positive and negative velocity components $v_z$, respectively), giving a net forward flux, i.e., in $+z$ direction, normal to the layer surfaces. In Fig. 1 this forward direction is vertically upwards, with *z* being the height above $\textrm{B}_0$. We assume steady-state isothermal conditions, gas and porous medium having the same temperature *T*. Provided that the gas is in thermal equilibrium above and below the layer at sufficient distance, the molecule velocities satisfy a Maxwell distribution with average molecule speed

10 $$\begin{aligned} {\overline{c}} = \sqrt{\frac{8RT}{\pi M}}, \end{aligned}$$

where $R=8.31\,\text {J/mol K}$ is the ideal gas constant and *M* the molar mass of the gas species. Near the lower surface, the molecules moving towards the layers satisfy the corresponding hemispherical Maxwell distribution ($v_z>0$), but the leaving molecules may have a different distribution. In particular they must have a different density if a steady-state net gas flow is to be maintained. However, it is plausible to assume that also the molecules moving backwards ($v_z<0$) have an overall hemispherical Maxwell distribution, albeit with a different density. The reason is that the preconditions of an isothermal process and diffuse reflection at the pore surface, with the surface area elements possessing an isotropic distribution of orientations, have these implications: (i) the energy distribution of the reflected molecules is the same as for the incident molecules, resulting in a Maxwell distribution of the molecule speed with the same average ${\overline{c}}$ in every direction; (ii) since the molecules ‘forget’ their direction of incidence at diffuse surface reflection, no direction is predominant among the reflected molecules when the forwards and backwards moving densities are not too different. In this case the vectorial velocity distribution of the molecules returning from the layer is practically Maxwellian. A similar argument holds for molecules entering the other side of the layer. Summing up, we apply a model of two hemispherical Maxwell velocity distributions at each cross section *z*, with two number densities $n^+(z)$ and $n^-(z)$ inside the pores for molecules with $v_z>0$ (moving forwards) and $v_z<0$ (moving backwards), respectively. This model was already successfully applied by Asaeda et al. [4] to derive $D^\textrm{K}$ for a monodisperse packing of spheres, the consequences of which are further studied in Sect. 4.

The partial densities $n^+(z)$ and $n^-(z)$ are defined as double the actual densities of the molecules moving in one direction, so that we can apply the familiar formula $J_\textrm{p}^\pm = n^\pm {\overline{c}}/4$ for the molecular flux $J_\textrm{p}^\pm$ inside the pores in the $\pm z$ direction (it amounts to the Hertz–Knudsen equation [19, 20] by means of (10) and the ideal gas law). The total number density is the average

11 $$\begin{aligned} n=(n^{+} + n^{-})/2. \end{aligned}$$

With the help of the split distribution $n^\pm (z)$ we can relate the transmission probability ${{\mathcal {T}}}$ and the half-transmission length $L_\textrm{h}$ to the Knudsen diffusion coefficient $D^\textrm{K}$. For symmetry reasons the total net flux *J* is parallel to the *z*-axis. It is composed of the unidirectional contributions $J^\pm$ of molecules moving forwards and backwards (in $+z$ and $-z$ direction, respectively)

12 $$\begin{aligned} J^\pm&= \epsilon \,\frac{n^\pm {\overline{c}}}{4}, \end{aligned}$$

13 $$\begin{aligned} J = J^{+}-J^{-}&= \epsilon \,\frac{(n^{+}-n^{-}){\overline{c}}}{4}. \end{aligned}$$

The factor $\epsilon$ accounts for the fact that inside the medium the gas can only move through the pores, wherefore the flux is reduced by the porosity. Here *J* is defined as the molar flow rate per cross section of the whole medium, whereas $J_\textrm{p}=J/\epsilon$ is the actual flux through the cross section of the void (open pores) in the medium. The vectorial flux can be expressed as a product of $D^\textrm{K}$ and the density gradient $\nabla n$ (Fick’s law for Knudsen diffusion), so we find the equality

14 $$\begin{aligned} J&= -D^\textrm{K}\,\frac{\textrm{d}n}{\textrm{d}z} = -D^\textrm{K}\,\frac{\textrm{d}}{\textrm{d}z}\left( \frac{n^{+}+n^{-}}{2}\right) . \end{aligned}$$

In the following the denotation $n^\pm _0 = n^\pm (0)$ and $n_0 = n(0)$ is used for brevity, and similarly for $z=L$. Thus, $n_0$ and $n_L$ are the total gas densities in the pores at the opposite surfaces of a layer ranging from $z=0$ to $z=L$. Utilizing the fact that *J*(*z*) is constant for a steady-state flow (in a vessel we have to assume constant cross-sectional area to ensure this condition), the last equation can be integrated over the layer height *L* to give

15 $$\begin{aligned} \frac{J\,L}{D^\textrm{K}} = -\frac{n^{+}+n^{-}}{2}\Big |_0^L = n_0-n_L. \end{aligned}$$

Under steady-state conditions, the fluxes of the molecules leaving the medium can be expressed in terms of the fluxes of the incident molecules and the probabilities of transmission ${{\mathcal {T}}}$ and reflection ${{\mathcal {R}}}=1-{{\mathcal {T}}}$ in the pores. Due to the proportionality (12) the corresponding relations can be written in terms of the respective densities

16 $$\begin{aligned} n_0^-&= {{\mathcal {R}}}\,n_0^{+} + {{\mathcal {T}}}\,n_L^{-}, \end{aligned}$$

17 $$\begin{aligned} n_L^+&= {{\mathcal {R}}}\,n_L^{-} + {{\mathcal {T}}}\,n_0^{+}. \end{aligned}$$

Substitution of these relations into the density-dependent terms of (13) and (15) yields

18 $$\begin{aligned} \frac{J\,L}{D^\textrm{K}}&=-\frac{n^{+}+n^{-}}{2}\Big |_0^L \nonumber \\&= \frac{1}{2}(n_0^{+}+{{\mathcal {R}}}\,n_0^{+}+{{\mathcal {T}}}\,n_L^{-})- \frac{1}{2}({{\mathcal {R}}}\,n_L^{-}+{{\mathcal {T}}}\,n_0^{+}+n_L^-) \nonumber \\&= {{\mathcal {R}}}\,(n_0^+-n_L^-), \end{aligned}$$

19 $$\begin{aligned} \frac{4\,J}{\epsilon \,{\overline{c}}}&= n^{+}-n^{-} \nonumber \\&= n_0^{+} - ({{\mathcal {R}}}\,n_0^{+} + {{\mathcal {T}}}\,n_L^{-}) \nonumber \\&= {{\mathcal {T}}}\,(n_0^{+} - n_L^{-}), \end{aligned}$$

$n^+ -n^-$ is independent of *z* due to the steady state of the flow, and the constant flux *J* can be eliminated from the above equations to obtain

20 $$\begin{aligned} D^\textrm{K} = \frac{\epsilon \,{\overline{c}} L\,{{\mathcal {T}}}}{4\, {{\mathcal {R}}}}. \end{aligned}$$

According to the definition (7) we finally arrive at the sought expression for the half-transmission thickness

21 $$\begin{aligned} L_\textrm{h} = \frac{4 D^\textrm{K}}{\epsilon \,{\overline{c}}}. \end{aligned}$$

These last two relations enable us to determine the transmission properties from models of the Knudsen diffusion coefficient. The most important examples are given in the following section.

## Expressions by models of Knudsen diffusion

Three well-known models for the Knudsen diffusion constant $D^\textrm{K}$ are used in this section to express the transmission properties in terms of the layer pore structure. The models were developed by Knudsen [1] (see also Epstein [21] for application to porous media), Derjaguin [3] and Asaeda et al. [4], respectively. We add another model for

layers of porous media with homogeneous and isotropic distributions of pore surface elements, by deriving the layer reflection probability and applying (20) to obtain $D^\textrm{K}$. In Sect. 5 the good concurrence with numerical simulations will prove it more accurate for sphere packings than the former models.

### Knudsen cylinder model

The first model is based on a representation of the pores as a set of slanted cylindrical tubes, which may have an average inclination with regard to the net flow direction. The model is based on Knudsen’s formula for the flow through a single cylinder of diameter $d_\textrm{c}$ [1]. Some adaptations are needed for porous media. Since the volume of the bundle of cylinders occupies only the void fraction $\epsilon$, the flux is reduced by the same factor. In addition, an inclination of the cylinders by an average angle $\theta$ results in a reduction of the pressure gradient along the cylinders by a factor $1/\tau =\cos \theta$ as well as an increase of the cylinder cross sections parallel to the layer planes by the factor $\tau =1/\cos \theta$. Both effects decrease the net flux by the same factor $1/\tau$. Thus, the diffusion coefficient of a bent cylinder bundle becomes [21]

22 $$\begin{aligned} D^\textrm{K} = \frac{\epsilon }{\tau ^2}\,\frac{d_\textrm{c}{\overline{c}}}{3}. \end{aligned}$$

The so-called tortuosity $\tau$ is typically between 1 and 3, but may also reach higher values. In a homogeneous and isotropic distribution of pieces of cylinder pores throughout the medium one would expect $\tau =\sqrt{3}$, but much larger values may occur in compactly packed beds [11], in particular when the grains are elongated or flat with the widest extensions lying normal to the net flow direction. The cylinder model shows the dependence on the cylindrical pore diameter and mean speed of gas molecules, but it does not fully account for the pore structure and the porosity of the medium (since the tortuosity depends on porosity as well).

### Derjaguin/Asaeda models

To overcome some of the disadvantages of the cylinder model Derjaguin [2] considered a packing of monodisperse spheres (republished as Derjaguin [3]), the results of which are shortly summarized in the following for its importance. Tracing the gas molecules gives a series of straight chords (rectilinear segments) between successive collisions with sphere surfaces. Adding chords and taking the average change of directions between successive segments into account, Derjaguin determined the diffusion coefficient in analogy to Brownian motion via the average square of the vectorial displacement $\textbf{d}$ gas molecules attain in time *t*,

23 $$\begin{aligned} \frac{D^\textrm{K}}{\epsilon } = \frac{\overline{\textbf{d}^2}}{6\,t} = \frac{{\overline{c}}}{6\,{\overline{\lambda }}} \left( \overline{\lambda ^2}-\frac{8}{13}{\overline{\lambda }}^2\right) . \end{aligned}$$

Here and in the following the bar (overline) expresses the average of the respective quantity (e.g., $\overline{\lambda ^2}$ is the average of the squared chord length, whereas ${\overline{\lambda }}^2$ is the square of the average chord length). In contrast to Derjaguin [3] we define the gas density as the moles per pore volume $V_\textrm{p}$ (and not per medium volume), wherefore the denominator $\epsilon$ appears on the left side of (23). This formula has been obtained under the assumption of diffuse reflection, for specular reflection the result is similar, with the second (subtracted) term in the parentheses omitted. The chord length distribution plays an essential role. According to (23) the first and second moments of the chord length distribution are sufficient to determine the diffusion coefficient for the Knudsen regime, provided diffuse or specular reflection occurs at the pore walls. A further simplification can be achieved by assuming that the molecule collision probability is independent of its past (Markov process), with $d\lambda /{\overline{\lambda }}$ being the probability of collision in the infinitesimal path element $d\lambda$, giving a homogeneous Poisson process with the mean chord length ${\overline{\lambda }}$ between successive collision events. With this assumption the chord lengths $\lambda$ obey the exponential distribution density $\exp (-\lambda /{\overline{\lambda }})/{\overline{\lambda }}$, with $\overline{\lambda ^2}=2{\overline{\lambda }}^2$. Assuming that the distribution of chords is homogeneous throughout the pore volume $V_\textrm{p}$ and the collisions are distributed uniformly over the pore surface $S_\textrm{p}$, the introduction of the specific surface $s_\textrm{p}=S_\textrm{p}/V_\textrm{p}$ of the pores is possible by means of [3]

24 $$\begin{aligned} {\overline{\lambda }} = \frac{4}{s_\textrm{p}} = \frac{2\,\epsilon \,d_\textrm{g}}{3(1-\epsilon )} \end{aligned}$$

with porosity $\epsilon$ and grain (sphere) diameter $d_\textrm{g}$. Substitution into (23) enables us to express $D^\textrm{K}$ in terms of $\epsilon$ and $d_\textrm{g}$

25 $$\begin{aligned} D^\textrm{K} = \frac{3\,\epsilon {\overline{\lambda }}\,{\overline{c}}}{13} = \frac{12\,\epsilon \,{\overline{c}}}{13\,s_\textrm{p}} = \frac{\epsilon ^2\,d_\textrm{g}\,{\overline{c}}}{3\Phi (1-\epsilon )} \end{aligned}$$

with the factor $\Phi =13/6$ as introduced by Asaeda et al. [4]. Here the specific surface of the pores, $s_\textrm{p}$, has been related to the specific surface of the solid, $s_\text {sol}$, via

26 $$\begin{aligned} s_\textrm{p} = \frac{1-\epsilon }{\epsilon }\,s_\text {sol} = \frac{6\,(1-\epsilon )}{d_\textrm{g}\epsilon }. \end{aligned}$$

A comparison of (25) with the Knudsen model shows the dependence of the tortuosity $\tau$ on the porosity $\epsilon$ of the medium.

The expression (25) was derived also by Asaeda et al. [4], but on the basis of a completely different approach. As mentioned in the former section a bi-hemispherical Maxwell distribution was considered for this purpose, with different densities for forwards and backwards moving molecules. The momentum loss due to the diffuse reflection on the spherical surfaces was calculated, resulting in the density gradient accompanied with the given flux. The authors arrived at the same equation (last term in (25)), with complicated integrals for the number $\Phi$, which they calculated to be 2.18. However, our numerical calculation of their integrals shows that $\Phi =13/6$ within quadrature accuracy, so full agreement with Derjaguin’s result is verified.

### Model based on reflection probability of sub-layers

For a homogenous packing we assume that in each thin sub-layer the distribution of pore surface elements is isotropic (no preferential surface directions). Therefore we calculate the reflection probability of such a sub-layer by summing up the back-scattered gas molecules over all pore surface elements, divided by the total number of incident molecules. This is achieved by first considering the fraction of molecules reflected by an infinitesimal element of the pore surface with outward normal $\textbf{n}$. For this purpose we have to define the reflection at the pore surface in mathematical terms. The diffuse reflection assumption used throughout this article is described by Knudsen’s reflection law, which is analogous to Lambert’s law for the diffuse reflection of photons at a plane surface. It states that the fraction of the molecules reflected into an infinitesimal solid angle $\textrm{d}\Omega _\textrm{r}$ around the direction $\textbf{e}_\textrm{r}$ is $\cos \alpha _\textrm{r}\,\textrm{d}\Omega _\textrm{r}/\pi$. Here $\alpha _\textrm{r}$ is the angle of reflection, i.e., the angle between the surface normal $\textbf{n}$ and the direction of departure $\textbf{e}_\textrm{r}$. This cosine law amounts to an isotropic angular distribution of the molecule flux (flow rate per unit projected area normal to the propagation direction) reflected into the unit solid angle.

Figure 2 illustrates the geometry of the molecule reflection at a collision point on the surface of a sphere. As in previous sections the layer is parallel to the *xy*-plane. To simplify calculations we choose the *x* axis in such a way that the *xz*-plane contains the normal of the tangential plane at the collision point. Let $\textbf{e}_\textrm{i}=\textbf{v}_\textrm{i}/|\textbf{v}_\textrm{i}|$ and $\textbf{e}_\textrm{r}=\textbf{v}_\textrm{r}/|\textbf{v}_\textrm{r}|$, with $\textbf{v}_\textrm{i}$ and $\textbf{v}_\textrm{r}$ being the velocity of incident and reflected molecules, i.e., $-\textbf{e}_\textrm{i}$ and $\textbf{e}_\textrm{r}$ are the directions of incidence and emission of a molecule when reflected at the surface. Further, let $\textrm{d}\Omega (\textbf{e}_\textrm{i})$ and $\textrm{d}\Omega (\textbf{e}_\textrm{r})$ be the corresponding infinitesimal solid angle elements around these directions. The negative velocity vectors $-\textbf{v}_\textrm{i}$ of the molecules incident on the given collision point with $v_z>0$ (‘forwards’ moving particles) build the shaded region in Fig. 2. It is defined by a solid angle range $\Omega (\textbf{n})$ for directions $-\textbf{e}_\textrm{i}=-\textbf{v}_\textrm{i}/|\textbf{v}_\textrm{i}|$, given by the two conditions $\textbf{e}_\textrm{i}\cdot \textbf{e}_z>0,$ and $-\textbf{e}_\textrm{i}\cdot \textbf{n}>0$, where $\textbf{e}_\textrm{z}$ denotes the unit vector in *z*-direction.

We utilize again the bi-hemispheric Maxwell distribution. The density of the molecules moving in a direction inside $\textrm{d}\Omega (\textbf{e}_\textrm{i})$ is $n^+ \textrm{d}\Omega (\textbf{e}_\textrm{i})/(4\pi )$. The projection of the surface element seen by the incident molecule is $-\textbf{e}_\textrm{i}\cdot \textbf{n}\,\textrm{d}A$. So the number of these molecules hitting the surface per unit time is $-\textbf{n}\cdot \textbf{e}_\textrm{i}\,\textrm{d}A\, {\overline{c}}\,n^+\,\textrm{d}\Omega (\textbf{e}_\textrm{i})/(4\pi )$, where ${\overline{c}}$ is the mean speed of the gas molecules. The total collision rate of forward moving molecules (i.e., with $v_z>0$) per unit area of the pore surface can be written

27 $$\begin{aligned} J_\textrm{i} = -\frac{{\overline{c}}\,n^+}{4\pi } \int _{\Omega (\textbf{n})} \textbf{n}\cdot \textbf{e}_\textrm{i}\, \textrm{d}\Omega (-\textbf{e}_\textrm{i}) = \frac{{\overline{c}}\,n^+}{4}\,\sin ^2(\theta /2). \end{aligned}$$

The main steps in the evaluation of this integral are given in A. As to be expected the collision rate $J_\textrm{i}$ of forwards moving molecules ($v_z>0$) depends only on the colatitude $\theta =\arccos (\textbf{n}\cdot \textbf{e}_z)$, i.e., the angle between the normal $\textbf{n}$ of the pore surface element and the *z*-axis, with $J_\textrm{i}=0$ when the surface normal points in *z*-direction.

We consider a uniform distribution of pore surface directions in each thin sub-layer. The fraction of molecules reflected by the layer is obtained by integrating the reflected molecules over all area elements $\textbf{n}\,\textrm{d}A$ present in the layer. Due to their isotropic distribution, we can express them by the specific surface $s_\textrm{p}$ of the pores. For this purpose we take into account that the pore volume $V_\textrm{p}$ of a thin sub-layer can be expressed by its thickness $\textrm{d}z$, the layer’s cross-section area *A*, and the porosity $\epsilon$,

28 $$\begin{aligned} V_\textrm{p}= &   \epsilon A \, \textrm{d}z \end{aligned}$$

29 $$\begin{aligned} \textrm{d}A(\textbf{n})= &   s_\textrm{p} V_\textrm{p} \frac{\textrm{d}\Omega (\textbf{n})}{4\pi }. \end{aligned}$$

The notations $\textrm{d}A(\textbf{n})$ and $\textrm{d}\Omega (\textbf{n})$ emphasize that the area and solid angle elements are associated with the surface normal direction $\textbf{n}$. $\textrm{d}A(\textbf{n})$ emits only a fraction of the incident molecules in directions $v_z<0$. Since the reflection is diffuse, the outgoing molecules are isotropically distributed in the pore hemisphere beyond the tangential plane at the respective surface point. This means that the surface emits the fraction $\textbf{n}\cdot \textbf{e}_\textrm{r}\,\textrm{d}\Omega (\textbf{e}_\textrm{r})/\pi$ into the solid angle $\textrm{d}\Omega (\textbf{e}_\textrm{r})$. So the rate of molecules reflected backwards at the surface element $\textrm{d}A(\textbf{n})$ (i.e., molecules incident with $v_z>0$ and scattered with $v_z<0$) is

30 $$\begin{aligned} J_\textrm{i} \textrm{d}A(\textbf{n}) \int _{\Omega (\textbf{n})} \textbf{n}\cdot \textbf{e}_\textrm{r}\, \textrm{d}\Omega (\textbf{e}_\textrm{r})/\pi = J_\textrm{i} \textrm{d}A(\textbf{n}) \sin ^2(\theta /2), \end{aligned}$$

where the same integral appears as in (27), but this time integration is over the set $\Omega (\textbf{n})$ of emission directions ${\textbf {e}}_\textrm{r}$, which are opposites to the set of ${\textbf {e}}_\textrm{i}$. Substitution of (27), (28), and (29), and integration over all surface elements yield the following reflected molecule flow rate:31where the integral in the second line is 2/3. Since the incident flow rate of molecules entering the layer from one side with $v_z>0$ is $A\epsilon {\overline{c}}n^+/4$, the reflection probability of the infinitesimal layer becomes $s_\textrm{p}\,\textrm{d}z/3$. On the other hand, it equals $\textrm{d}z/L_\textrm{h}$ in virtue of equation (8) for infinitesimal thickness $L=\textrm{d}z$. Thus, we obtain a formula for the half-transmission length

32 $$\begin{aligned} L_\textrm{h} = \frac{3}{s_\textrm{p}}. \end{aligned}$$

By means of the relation (21) we finally yield another model for the Knudsen diffusion coefficient

33 $$\begin{aligned} D^\textrm{K} = \frac{\epsilon \,{\overline{c}}L_\textrm{h}}{4} = \frac{3\,\epsilon \,{\overline{c}}}{4\,s_\textrm{p}} = \frac{{\overline{c}}\,d_\textrm{g}\,\epsilon ^2}{8\,(1-\epsilon )}. \end{aligned}$$

This result has been entirely derived on the basis of probabilistic considerations, which is in contrast to Asaeda et al. [4] who applied momentum conservation to the reflection process. The preconditions of the layer model are diffuse reflection at pore surfaces, constant temperature throughout the sample (and gas volume), and a homogeneous and isotropic distribution of pore surface elements in each thin sub-layer on a lateral scale much greater than the sphere diameters.

The probability model (33) is similar to the formula (25) derived by Asaeda et al. [4], which is a simplification of the more general formula (23) derived by Derjaguin [2]. Our result (33) is by a constant factor 13/16 smaller than (25). Since the numerical simulations confirm the higher accuracy of our result (33), we can conclude that the simplification that implies the Asaeda formula (25) from the more general Derjaguin formula (23) does not hold well for random sphere packing. That is, the chord lengths $\lambda$ do not obey the distribution density $\exp (-\lambda /{\overline{\lambda }})/{\overline{\lambda }}$, and $\overline{\lambda ^2}=2{\overline{\lambda }}^2$ is an estimate that is not quite accurate (as shown in Sect. 5 leading to an error of about 20 % for ideal sphere packings). Table 1 summarizes the relation between the different models when expressed as half-transmission thickness $L_\textrm{h}$.

**Table 1:** Half-transmission thickness of homogeneous layers of packed spheres, as related to different models of $D^\textrm{K}$ via (21)

Model	Derivation principle		(21)
Knudsen	Cylinder model		(34)
This article	Layer reflection	$\displaystyle \frac{3}{s_\textrm{p}} = \frac{d_\textrm{g}\epsilon }{2(1-\epsilon )}$	(35)
Derjaguin	Squared displacement	$\displaystyle \frac{2}{3\,{\overline{\lambda }}} \left( \overline{\lambda ^2}-\frac{8}{13}{\overline{\lambda }}^2\right)$	(36)
Asaeda	Momentum balance	$\displaystyle \frac{48}{13\,s_\textrm{p}} = \frac{8\,d_\textrm{g}\epsilon }{13(1-\epsilon )} = \frac{12{\overline{\lambda }}}{13}$	(37)

The last line can be obtained from the previous line by the above-discussed approximation of the path length distribution. The equations in Table 1 reveal that the half-transmission length $L_\textrm{h}$ is similar to the mean chord length. It is of the same order of magnitude as the equivalent cylinder diameter if the tortuosity is not too high, which depends on the porosity as revealed by comparing (34) with (35) and (37). When equating $d_\textrm{c}=d_\textrm{g}$ the tortuosity factor alone is used to represent the influence of the grain arrangement (and so the pore structure):

38 $$\begin{aligned} \tau ^2 = \frac{13(1-\epsilon )}{6\,\epsilon }\,q. \end{aligned}$$

Here $q=16/13$ or $q=1$ when (35) or (37), respectively, is used as a reference. The comparison of the Knudsen cylinder model with other models as reference requires different *q* values. The additional correction factor *q* can also be chosen in accordance with results from experiments or simulations. The advantage of expressing $\tau$ in terms of (38) is that it represents well the main porosity dependence for realistic sphere packings (in agreement with the discussed models), leaving only a minor porosity dependence of the correction factor *q* (an example is given below). According to formula (35), $L_\textrm{h}$ agrees with the sphere diameter for packings of porosity $\epsilon \approx 2/3$. A considerable increase of porosity above this value can make the medium much more permeable to gas flow, with a substantial amount of molecules advancing much deeper than a grain size into the layer ($L_\textrm{h}>d_\textrm{g}$). This situation occurs in comet nucleus material where the low gravity allows very high porosities of 70-75%, which may even reach above 80% at certain places [22].

The model (37) by Asaeda et al. [4] was derived under the precondition that all spheres have the same diameter (monodispersity). In contrast, formula (36) by Derjaguin [2] and also the above result (32) are valid for polydisperse packings as well. The Derjaguin model as well as our derivation for the layer model (when expressed in terms of the specific surface $s_\textrm{p}$) do not assume anything about the grain size and shape, apart from the precondition of an isotropic and homogeneous distribution of surface elements of the pore walls, and that the emission directions of successive collisions are independent of each other. Of course, the assumption of homogeneity amounts to a good mixing of the spheres of all sizes in a polydisperse packing. For this case (35) can be generalized by adding the contributions of all sorts of spheres to the specific surface. So (26) implies

39 $$\begin{aligned} s_\textrm{p} = \frac{6(1-\epsilon )}{\epsilon } \sum _m n_{m}\,d _{m}^2 \left( \sum _m n_{m}\,d _{m}^3 \right) ^{-1}, \end{aligned}$$

where the sum goes over all sorts of spheres with $n_{m}$ being the number density and $d _{m}$ the diameter of the sort *m*.

In practice, we have to expect that realistic packings (even for spherical grains) are not perfectly homogeneous and depend on the creation process. This holds even more for angular grains. A variation of the spatial distribution of grain diameters can occur due to sample vibration under gravity, causing segregation (Brazil nut effect). Dependent on the packing process, the pore space may include irregularly distributed large cavities or constrictions, and it may contain anisotropic structures on a scale larger than the average chord length of gas molecule motion. In consequence, certain regions may be easier accessible to the gas flow than others, and anisotropy may result in direction-dependent permeability of the medium. Such effects cannot be represented well by the discussed models. An attempt to account for some of these effects is often made by introducing a suitable tortuosity. For instance, Güttler et al. [23] have recently shown by DSMC simulations for packings of steel spheres that the introduction of a porosity-dependent correction factor $q=1.6-0.73\,\epsilon$ in (38) substantially improves the agreement of the model with the simulation results. Of course, a location- and direction-dependent character of a sample cannot be represented by this heuristic approach.

## Numerical simulations

### Overview of methods

For the assessment of the validity of the formulas (21) and (35)–(37) we perform numerical simulations for sphere packings of different porosities but constant grain diameters. Since solvers for fluid flow are not applicable in the Knudsen regime, we have to resort to programs which follow the molecules (or group of molecules building a fictive particle) as they travel through the pore space of the medium. Molecular Dynamics (MD) solvers [24] can be used to model the interaction of gas molecules with each other and with the pore surface in detail. Since we focus on diffuse reflection and large number of molecules meandering through a complex pore geometry, MD would take extremely large computation times. The Lattice Boltzmann Method (LBM) can be used in the transition regime to simulate the behavior of a gas molecule ensemble passing the layer [25]. However, it is based on a lattice discretization and therefore not the best choice when the possible trajectories of molecules moving through the layer are to be determined. For these reasons we exclude MD and LBM solvers.

The diffuse collisions at the pore wall of the solid matrix are not deterministic but obey a stochastic rule. So the molecule trajectories are random walks, composed of chords between successive collisions with the pore wall. Therefore, the first approach for validation of the analytical models is a specific Random Walk (RW) algorithm. Actually it is a ‘Test Particle Monte Carlo’ method which has been successfully applied for years in the context of gas transport in cometary material [14]. Details on the theoretical background and algorithm can be found in the pertaining literature [26, 27], where also further references are provided. In the following we continue to refer to it as Random Walk (RW) due to its nature and the fact that each single particle is followed along its erratic path through the pores. The advantage of this approach (compared to the other applied method) is that it works with sphere packings, handling the spherical geometry precisely, without the need of representing the sphere surface by an appropriate mesh. The RW program is applied to calculate the penetration depth of molecules into the porous layer, giving directly the transmission probability ${\mathcal {T}}(z)$ as a function of the layer thickness *z*.

As a second numerical technique we apply the Direct Simulation Monte Carlo (DSMC) program SPARTA^1^ [28]. It is used in two ways: first, to verify the penetration depth of gas molecules, and second, to determine the density distribution of the gas flow through the packing under steady-state conditions. The DSMC is dedicated to the simulation of rarified gas flows, based on an algorithm for surrogate molecules, each of which represents a huge number of real molecules. The theoretic justification of the approach and its implementation have been studied for decades. An in-depth description is given by the main developer of DSMC [6]. The DSMC can also be applied in the transition regime, where, in addition to Knudsen diffusion, viscous flow contributions start to play a role. However, here we focus on the Knudsen diffusion by switching off intermolecular collisions and only allowing for collisions at the pore surface. In contrast to the applied RW program, the spheres are represented as triangle meshes, which requires more preprocessing of the models.

### Packing geometry and preparation

**Fig. 3:**
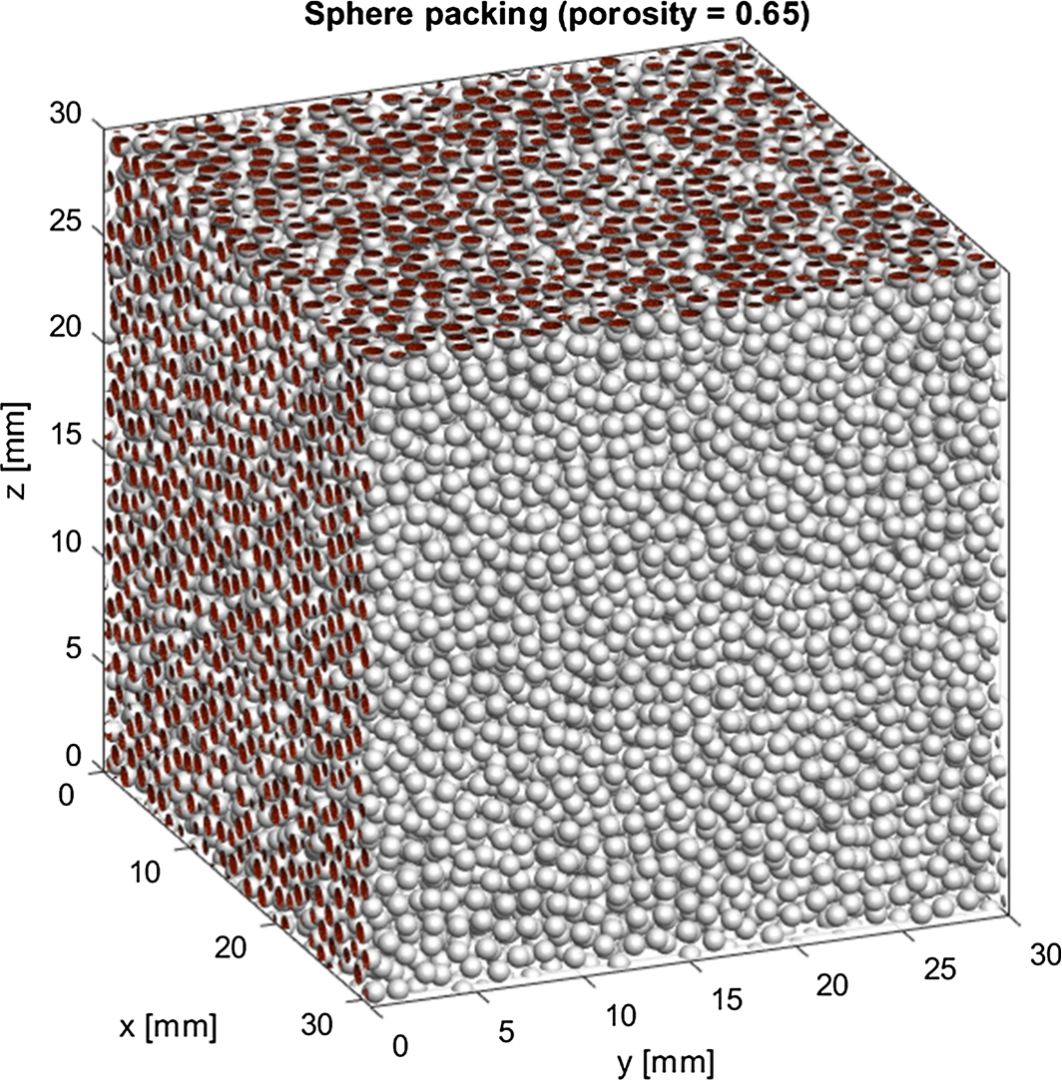
Grain packing of 1 mm diameter spheres in a cube of 30 mm side length, as used for the simulation of a layer with porosity 0.65. Sphere cuts at the box boundaries are colored red (for better visibility cuts are omitted at the side facing the observer)

Simulation samples of fixed porosities are generated in a cuboid box, as illustrated in Fig. 3 for the sample with $\epsilon =0.65$. In the simulations the gas molecules enter the box at the bottom ($z=0$) and transmitted molecules leave at the top ($z=30\,\text {mm}$) of the box. At the vertical sides periodic boundary conditions are applied. The spheres projecting beyond the sample box are cut off, leaving cuts shown as circular red disks at the box boundary. At the side facing the observer the cuts are omitted for better visibility of the packing.

**Fig. 4:**
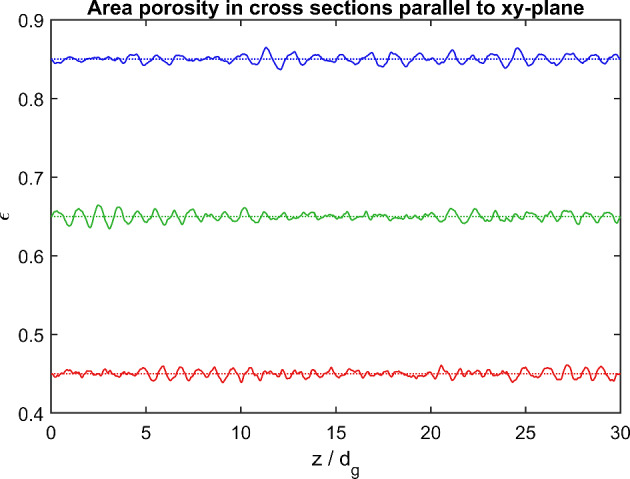
Areal porosity (solid lines) for cross sections at height *z* in the simulated samples with nominal porosity 0.45, 0.65, and 0.85 (dash lines)

Special care is taken to prepare packings with random but uniformly distributed spheres. This means that the porosity undergoes only slight changes on a scale much larger than a single sphere. Figure 4 shows the area porosity (the area of the void part divided by the total area of a layer cross section) as a function of the height *z* in the simulated samples. To avoid significant variation of porosity near the wall of the sample box, the packing is constructed with periodic conditions at opposite box boundaries, so that an infinite layer is well simulated when the simulation domain is about 20 or more sphere diameters wide. All conditions are achieved by the packing routines developed by Vasili Baranov, which he provides via a github repository.^2^ Three packings of 1 mm spheres are used, with filling factors 0.55, 0.35, and 0.15, covering a wide porosity range ($\epsilon =0.45$, 0.65, and 0.85). Since the sphere diameter of 1 mm and the size of the simulated sample (cube-shaped box of 30 mm side length) are always the same, different number of spheres need be used to obtain the sought porosities, which amounts to about 30000, 18000, and 8000 for the respective packing.

The following considerations led to the choice of the millimeter sphere diameter: At a fixed gas temperature the studied Knudsen diffusion models can be well transferred to a scaled geometry (where all grain radii and their position vectors are multiplied by the same scale factor, which keeps the porosity constant): The Knudsen diffusion constant $D^\textrm{K}$ and the half-transmission thickness $L_\textrm{h}$ have to be multiplied by the scale factor, whereas the transmission and reflection probabilities, ${\mathcal {T}}$ and ${\mathcal {R}}$, stay the same. Therefore the choice of the sphere size for the simulations is not essential when only the molecular diffusion (Knudsen diffusion) is investigated. The results are applicable, as well, to mesoporous materials or larger grains. The only precondition is that the gas densities stay in the Knudsen regime. For instance, dust on comets covers the whole mentioned size range. Because of the low gas pressure on the comet, the Knudsen regime prevails in pores up to about millimeter size. This is of particular interest in the context of dust agglomerates in near-surface layers, which are of great importance for the thermal balance and outgassing of the comets [30, 31]. Packing of millimeter size are also very suitable for laboratory measurements to study the Knudsen diffusion because of handling and safety aspects. This holds especially for spherical grains, since the sphericity cannot be guaranteed for much finer fractions due to the production process. Of course one has to take care that the Knudsen regime prevails in the experiments, which requires a gas pressure below some Pa.

The generated packed samples were imported into SPARTA/DSMC as surface files of stl-format. The stl triangle meshes were constructed in MATLAB^3^ from the output files (containing sphere centers) of the Baranau routines. Thus, a further preprocessing step was necessary to represent each sphere by surface segmentation of sufficient resolution (so as to avoid porosity errors). After import, the surfaces of spheres projecting beyond the simulation box are cut at the simulation boundary (cube surface). In contrast to SPARTA, the RW algorithm is based on ideal sphere geometry, so just the definition of the sphere centers and their diameter is needed as input. Either method (RW and DSMC) was used to obtain the height *z* the gas molecules reach when incident on the $z=0$ face of the box.

**Fig. 5:**
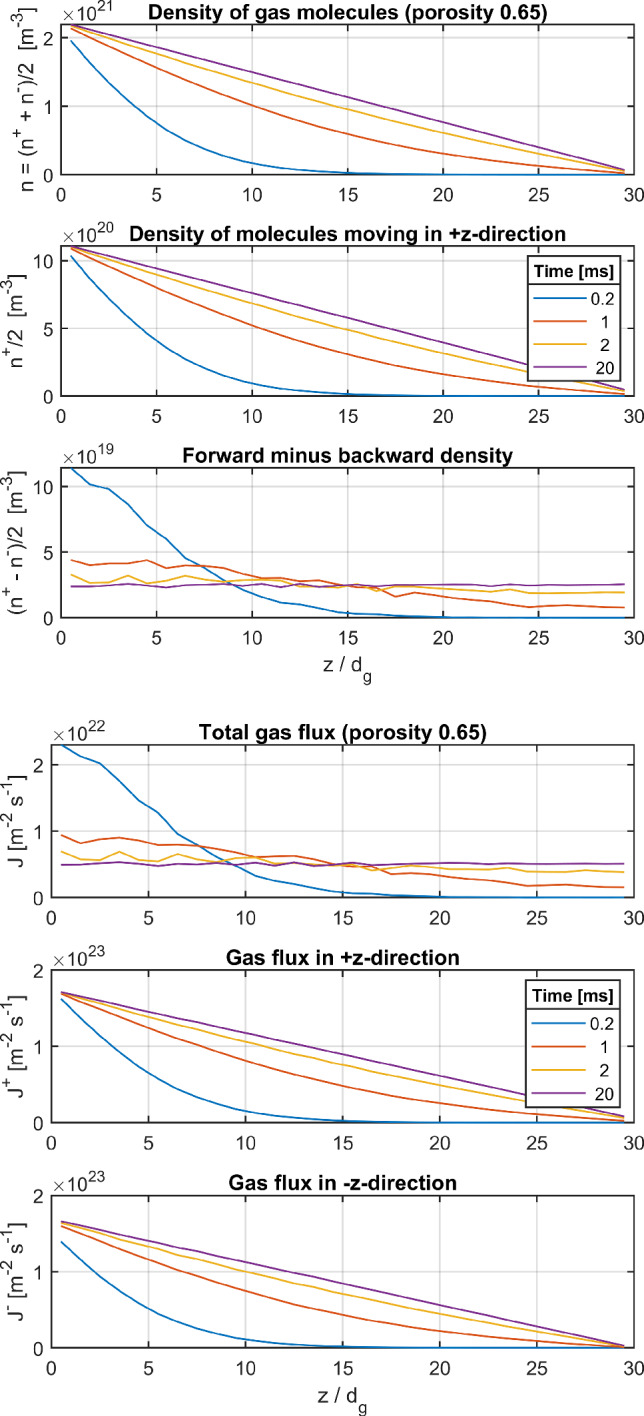
Density and flux evolution of the simulated gas flow through the sample of porosity 0.65 until attainment of steady-state conditions after about 20 ms

### Simulation results

**Fig. 6:**
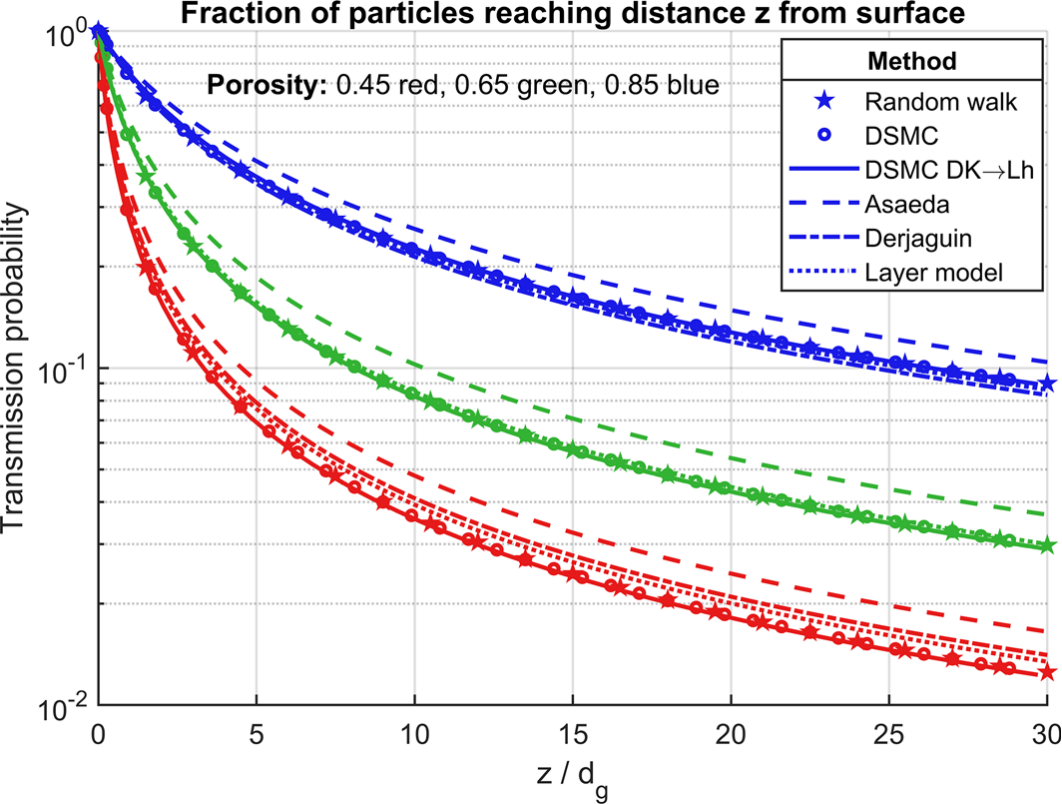
Transmission probability of a layer as a function of thickness *z* measured in grain diameters $d_\textrm{g}$ (equal to the fraction of molecules reaching height *z* from the entrance surface). The results of different models, as explained in the main text, are shown for three different porosities

As mentioned above, first RW as well as DSMC were used to determine the height particles reach in the sample. A large number of molecules (several millions) was used for this study step, incident to the bottom of the simulation box with Maxwellian velocity distribution. The fraction of molecules reaching height *z* is equal to the transmission probability ${\mathcal {T}}(z)$ of a layer of thickness *z*. The dependence of ${\mathcal {T}}$ on *z* obtained in this manner is exhibited in Fig. 6, where stars and circles mark RW and DSMC results, respectively. Though the two approaches are based on totally different algorithms, their results agree very well, confirming their accuracy.

In a second study step with DSMC, a constant incident flux of gas molecules is prescribed at $z=0$, starting at time $t=0$ with no gas in the sample. The applied gas species is $\text {N}_2$, with a Maxwellian velocity distribution of incident molecules at a temperature of 296 K. Since the intermolecular collisions were switched off in the SPARTA script to force Knudsen diffusion, the applied pressure is irrelevant. Precisely speaking, the resulting density and flux distributions depend linearly on the pressure $p_0$ at the entrance (bottom side of the sample box), giving the same Knudsen diffusion coefficient independent of $p_0$. The simulation was run until a steady-state flow was achieved within the sample. The evolution of the density and flux distribution in the sample with $\epsilon =0.65$ are plotted in Fig. 5. The density of the positive flux component, $n^+(z)/2$, and the difference of forward and backward moving gas components, $(n^+(z)-n^-(z))/2$, are shown in addition to the total density $n(z)=(n^+(z)+n^-(z))/2$. Similarly, the flux components $J^\pm (z)$ are shown in addition to the total net flux $J(z)=J^+(z)+J^-(z)$. The evolution of the density and flux can be clearly seen. Almost no gas has reached the upper half of the sample after about 0.2 ms. At $t=2\,\text {ms}$ the gas has spread well all over the sample, and the flux undergoes only a small variation from the entrance to the outlet side of the box. At $t=20\,\text {ms}$ the transient process has well achieved its steady-state limit, with a linear drop of the density across the sample and a constant flux throughout the whole sample.

From the resulting gas density gradient $\textrm{d}n/\textrm{d}z$ and flux *J* the Knudsen diffusion constant $D^\textrm{K}$ is calculated my means of (14). By applying (21) the half-transmission thickness $L_\textrm{h}$ is obtained, which finally is used in (9) to draw the transmission probability ${\mathcal {T}}(z)$. The corresponding curves are shown in Fig. 6 as solid lines (denoted as ‘DSMC DK$-\!>$Lh’ in the annotation). The results of this steady-state DSMC approach agree perfectly with the direct determination of the transmitted molecule fractions as obtained above with RW as well as DSMC (circle and star markers in Fig. 6). So the simulations provide a very good validation of the relation (21) between $D^\textrm{K}$ and $L_\textrm{h}$.

### Comparison of simulations with analytical models

In order to assess the accuracy of the analytical $D^\textrm{K}$ models, (9) is applied to the formulas (35)–(37), and the resulting functions ${\mathcal {T}}(z)$ are plotted in Fig. 6). So a comparison of the analytic models with the simulations is possible.

As already discussed by Asaeda et al. [4], their model overestimates $D^\textrm{K}$ significantly in a systematic manner (approximately by a constant factor of 1.4). Therefore they introduced the tortuosity correction factor *q* for compensation, just as usually done for the cylindrical Knudsen model (however, the two definitions $\tau ^2$ and *q* are different, since $\tau ^2$ contains the main part of the porosity dependence as discussed after (38)).

The relations (36) by Derjaguin and (35) derived in the previous section agree much better with the numerical results. On the average (35) performs best: At porosities above 0.6 the corresponding $D^\textrm{K}$ results deviate only about 3 %. The agreement deteriorates when approaching low porosity (high compaction), reaching about 10 % relative deviation at $\epsilon =0.45$.

The general Derjaguin model (36) has been calculated, as well, from the DSMC simulations. For this purpose, all chords (free paths $\lambda$ between surface collisions) of the gas molecules are collected, for which the corresponding mean values ${\bar{\lambda }}$ and $\overline{\lambda ^2}$ are calculated and used in (36). This approach yields much better results than the Asaeda model (35). Since the latter can be derived from the former by approximating $\overline{\lambda ^2}=2{\bar{\lambda }}^2$, we can conclude that this equation is not well satisfied for the investigated packings.

Though the agreement of the models (36) and (35) with the numerical simulations is quite well, we have to state that in realistic packings the measured $D^\textrm{K}$ values are still lower, since in reality grains have angular surfaces, and even spherical glass beads have slightly uneven and rough surfaces. As a consequence, the specific surface of the pores is higher than the nominal value determined from the sphere diameters. So gas molecules undergo more collisions in the pores when they pass a layer of given porosity. This may but need not change the transmission probability through the layer. For instance tiny traps on the surface of very rough grains may cause a trapped gas molecule to collide many times before leaving the trap without changing the overall probability of passing the whole layer [32]. The presented theoretical considerations in Sect. 2 are only applicable if the cross-sectional area porosity is sufficiently constant across the sample. However, this condition can be effectively violated when irregularly distributed tiny traps are present in a finite sample. In particular, when working with aggregates of finer grains, the pore space inside the aggregates can be regarded as traps, which may have only negligible influence on the transmission probability, although the gas molecules may stay (move) for a long time inside the aggregates. In order to quantify these effects better, the study of gas flow through hierarchical pore structures has attracted considerable attention recently [30, 31, 33].

## Conclusion

The presented analytical approach facilitates the characterization of steady-state isothermal gas flow through plane layers of porous materials, when the distribution of pores is homogeneous on a scale greater than the typical pore size. It is assumed that the gas molecule reflection at pore surfaces is diffuse and no adsorption takes place. By considering the transmission probability ${{\mathcal {T}}}$ and reflection probability ${{\mathcal {R}}}=1-{{\mathcal {T}}}$ of layer partitions it was shown that ${{\mathcal {R}}}/{{\mathcal {T}}}$ is an additive quantity. In other words, its value equals the sum of the respective quantities ${{\mathcal {R}}}_i/{{\mathcal {T}}}_i$ of a subdivision into *n* sub-layers $i=1\dots n$ along the net flux direction *z*. By taking the limit to infinitesimal sub-layers, it is recognized that $L_\textrm{h}=L{{\mathcal {T}}}/{{\mathcal {R}}}$ is independent of the layer thickness *L* and represents a property of the medium itself, which is revealed as the layer thickness for which half the incident gas molecules traverse the layer (the other half being reflected).

By applying a bi-hemispherical Maxwell distribution, splitting the gas density into the unidirectional components of forward and backward moving molecules, the link between gas density and flux components is made. This model was used by Asaeda et al. [4] to calculate the Knudsen diffusion constant $D^\textrm{K}$ of sphere packings. In the present article it is applied to relate ${{\mathcal {T}}}$ and $L_\textrm{h}$ to $D^\textrm{K}$. This relation enables one to calculate the transmission properties of layers (given by ${{\mathcal {T}}}$) on the basis of $D^\textrm{K}$ models, or vice versa to find $D^\textrm{K}$ from measured fractions ${{\mathcal {T}}}$ of transmitted gas molecules. The relation proves useful in technical applications and laboratory experiments, but also in the evaluation of space science observations (comet outgassing), when one of the quantities can be determined.

By a further application of this stochastic approach to the reflections of gas molecules at the surface area elements in a thin sub-layer, an analytic model of $D^\textrm{K}$ is derived which differs from the former model by Asaeda et al. [4] by a constant factor 13/16. Numerical simulations with Random Walk and Direct Simulation Monte Carlo programs verify that the model derived here is more accurate than the former models. On the average, our layer model performs even better than Derjaguins general formula, which is based on the first two moments of the distribution of chord lengths (length between successive collisions of the gas molecules with the pore surface). The simulations further confirm the validity of the obtained relation between ${{\mathcal {T}}}$, $L_\textrm{h}$, and $D^\textrm{K}$. In order that the presented probabilistic approach is valid, cross-sectional areas must be large with regard to grain or pore size, since the transmission probability must be independent of *x* and *y* (location on the plane normal to the net flux) on a macroscopic scale. More precisely, the probability definitions must be regarded as averages over all directions of incidence, and over areas much greater than the grain or pore size.

While the stochastic layer approach generally serves a better understanding of gas transport processes in porous media, it is of direct practical use, too. Since analytical formulas for the $D^\textrm{K}$ are often part of gas flow studies in the Knudsen regime, our models can contribute to a more accurate approximation of real scenarios in such cases [34, 35]. Similarly, the relation between $L_\textrm{h}$ and $D^\textrm{K}$ is a valuable tool, since it enables us to compare the results of simulations based on the former quantity with experiments and observations that are interpreted by the latter quantity. These merits are also motivation to generalize the models to more complex scenarios. For instance, an extension of the formalism to stratified media, where the medium properties (porosity and half-transmission thickness) change with height in the layer, is conceivable when the other mentioned preconditions are maintained. Corresponding work is planned in view of the many applications, e.g., laboratory measurements of granular samples and complex mesoporous materials, and gas transport in comet surface layers (which is not only relevant for the thermal evolution but also for dust entrainment of outgassing volatiles [36]). Of course, certain effects can scarcely be treated analytically and must be left to numerical simulations, e.g., irregular inhomogeneity or anisotropy of the pore distribution in the medium, especially irregular hierarchical structures with various sorts and dimensions of pores, which are of great interest in many fields.

## Appendix A: Evaluation of the applied solid angle integral

The integral appearing in (27) is considered. The domain of integration is the solid angle range of all points in the shaded region in Fig. 2. It consists of all directions $\textbf{e}_\textrm{i}$ satisfying the relations $\cos \alpha _\textrm{i}=-\textbf{n}\cdot \textbf{e}_\textrm{i}>0$ and $\textbf{e}_z\cdot \textbf{e}_\textrm{i}>0$, where $\textbf{e}_z$ is the unit vector in *z* direction and $\alpha _\textrm{i}$ is the angle of incidence, i.e., the angle between the direction of incidence $-\textbf{e}_\textrm{i}$ and the unit surface normal $\textbf{n}$ pointing into the pore space. For the evaluation of the integral expedient spherical coordinates $(\delta _\textrm{i},\psi _\textrm{i})$ are introduced to represent the unit vector $-\textbf{e}_\textrm{i}$, where the *y*-axis is the polar axis, $\delta _\textrm{i}$ is the colatitude (angle between $\textbf{e}_y$ and $-\textbf{e}_\textrm{i}$), and $\psi _\textrm{i}$ is the azimuth measured from the $+x$ axis towards $-z$. Analogous spherical coordinates $(\delta ,\psi )$ are defined for the surface normal $\textbf{n}$. For simplification we assume that the coordinate frame is turned in such a way that the surface normal lies in the *xz* plane ($\delta =\pi /2$, $\psi =\theta -\pi /2$) with $|\psi |<\pi /2$. Here $\theta$ is the angle between the surface outward normal and the *z*-axis, as shown in Fig. 2. Thus,

A.1 $$\begin{aligned} -\textbf{e}_\textrm{i}= &   \left( \begin{array}{c} \sin \delta _\textrm{i}\cos \psi _\textrm{i}\\ \cos \delta _\textrm{i} \\ -\sin \delta _\textrm{i}\sin \psi _\textrm{i} \end{array} \right) \end{aligned}$$

A.2 $$\begin{aligned} \textbf{n}= &   \left( \begin{array}{c} \cos \psi \\ 0 \\ -\sin \psi \end{array} \right) = \left( \begin{array}{c} \sin \theta \\ 0 \\ \cos \theta \end{array} \right) . \end{aligned}$$

Substituting the solid angle $\textrm{d}\Omega (-\textbf{e}_\textrm{i})= \sin \delta _\textrm{i}\,\textrm{d}\delta _\textrm{i}\,\textrm{d}\psi _\textrm{i}$ the sought integral becomes

A.3 $$\begin{aligned}  &   -\int _{\Omega (\textbf{n})} \textbf{n}\cdot \textbf{e}_\textrm{i}\, \textrm{d}\Omega (-\textbf{e}_\textrm{i}) \nonumber \\  &   \quad = \int _0^\pi \textrm{d}\delta _\textrm{i}\,(\sin \delta _\textrm{i})^2 \int _0^{\theta } \textrm{d}\psi _\textrm{i}\, \sin (\theta -\psi _\textrm{i}) \nonumber \\  &   \quad = \frac{\pi }{2}\left( 1-\cos \theta \right) = \pi \sin ^2(\theta /2), \end{aligned}$$

where the azimuth range $0=\max (0,\psi -\pi /2)<\psi _\textrm{i}<\min (\psi +\pi /2,\pi )=\theta$ led to the limits of the inner integral in the second line.

**Table 2:** Symbols for most important quantities and constants

Symbol	Description
*T*	Temperature of gas and medium
$d_\textrm{p}, d_\textrm{c}$	Pore size, cylinder diameter
$d_\textrm{g}$	Grain size (sphere diameter)
$S_\textrm{p}$, $V_\textrm{p}$	Surface and volume of pores
$s_\textrm{p}$	Specific surface of pores ($=S_\textrm{p}/V_\textrm{p}$)
$s_\text {sol}$	Specific surface of solid matrix ($=s_\textrm{p}\,\epsilon /(1-\epsilon )$)
$\epsilon$	Porosity
${\overline{c}}$	Mean speed of gas molecules
*M*	Molar mass of gas species
*J*	Molecular flux $[\mathrm {mol/(m^2\,s)}]$ through layer cross section
$J_\textrm{p}$	Molar molecular flux through pore cross section ($=\!J/\epsilon$)
$n^\pm /2$	Density $[\mathrm {mol/m^3}]$ of molecules with $v_z\gtrless 0$
${{\mathcal {T}}}$	Layer-pores transmission probability
${{\mathcal {R}}}$	Layer-pores reflection probability
*R*	Ideal gas constant
$L_\textrm{h}$	Half-transmission thickness (for ${{\mathcal {T}}}={{\mathcal {R}}}=1/2$)
$D^\textrm{K}$	Knudsen diffusion coefficient
$\tau$	Tortuosity
${\overline{\lambda }}$	Mean chord length
$\Phi$	13/6 as introduced by [4]

## Footnotes

1 SPARTA (Stochastic PArallel Rarefied-gas Time-accurate Analyzer) is a parallel DSMC for simulations of rarified gases, primarily developed at Sandia National Laboratories, US Department of Energy (DOE), http://sparta.sandia.gov.

2 The program package is available at https://github.com/VasiliBaranov/packing-generation, with certain underlying principles discussed in Baranau and Tallarek [29].

3 MATLAB numeric and symbolic math programming language/environment by MathWorks.
